# Supporting Adolescent Engagement with Artificial Intelligence–Driven Digital Health Behavior Change Interventions

**DOI:** 10.2196/40306

**Published:** 2023-05-24

**Authors:** Alison Giovanelli, Jonathan Rowe, Madelynn Taylor, Mark Berna, Kathleen P Tebb, Carlos Penilla, Marianne Pugatch, James Lester, Elizabeth M Ozer

**Affiliations:** 1 Department of Pediatrics University of California, San Francisco San Francisco, CA United States; 2 Department of Computer Science North Carolina State University Raleigh, CA United States; 3 Office of Diversity and Outreach University of California, San Francisco San Francisco, CA United States

**Keywords:** digital health behavior change, adolescent, adolescence, behavior change, BCT, behavioral intervention, artificial intelligence, machine learning, model, AI ethics, trace log data, ethics, ethical, youth, risky behavior, engagement, privacy, security, optimization, operationalization

## Abstract

Understanding and optimizing adolescent-specific engagement with behavior change interventions will open doors for providers to promote healthy changes in an age group that is simultaneously difficult to engage and especially important to affect. For digital interventions, there is untapped potential in combining the vastness of process-level data with the analytical power of artificial intelligence (AI) to understand not only how adolescents engage but also how to improve upon interventions with the goal of increasing engagement and, ultimately, efficacy. Rooted in the example of the INSPIRE narrative-centered digital health behavior change intervention (DHBCI) for adolescent risky behaviors around alcohol use, we propose a framework for harnessing AI to accomplish 4 goals that are pertinent to health care providers and software developers alike: measurement of adolescent engagement, modeling of adolescent engagement, optimization of current interventions, and generation of novel interventions. Operationalization of this framework with youths must be situated in the ethical use of this technology, and we have outlined the potential pitfalls of AI with particular attention to privacy concerns for adolescents. Given how recently AI advances have opened up these possibilities in this field, the opportunities for further investigation are plenty.

## Introduction

Adolescence is characterized by an increase in risky behaviors that contribute to leading causes of injury and death [1]. Preventive interventions that provide education and counseling are recommended by the American Academy of Pediatrics to reduce risky behaviors and promote adolescent health [1-4]. However, adolescents’ access to such services is often low [5-7]. At the same time, adolescents use technology at higher rates than any other age group [8,9] and endorse comfort with technology in health care settings [10,11]. As such, the field of adolescent health has begun to turn to technology to improve the reach and effectiveness of preventive interventions for adolescent health behavior change [5,12-21]. While this early work is promising, there remains a significant untapped opportunity to leverage digital health behavior change interventions (DHBCIs) for adolescent health [5,14,16].

DHBCIs are designed to promote behavior change through many of the same mechanisms as traditional behavior change interventions, such as enhancing motivation and engagement, building skills, and increasing knowledge and self-efficacy [22-25]. Engagement, in particular, is a key predictor of intervention effectiveness [25-27] and is an area where the affordances of digital technology may render DHBCIs advantageous over traditional behavior change interventions [19]. At the same time, adolescent engagement and its association with intervention effectiveness are not fully understood [28]. Several frameworks have been generated to illuminate and measure engagement with DHBCIs [29-32], but they stop short of elucidating the role of rapidly advancing computing technologies in this arena.

## Artificial Intelligence and Machine Learning Can Enhance Adolescent DHBCIs

Recent advances in artificial intelligence (AI), and particularly machine learning, show significant promise for enriching the design and effectiveness of DHBCIs for adolescent behavior change [33]. AI is creating new opportunities for technology-based personalization and increased responsiveness to users across a range of sectors, including education [34,35], entertainment [36], and increasingly, health care [37]. Advances in AI also hold significant potential for better understanding and supporting adolescent engagement with DHBCIs [33]. A key source of engagement data and an enabler of AI functionalities in DHBCIs is *interaction trace log data*. As adolescents engage with a DHBCI, a detailed record of their moment-to-moment interactions with the technology can be captured (eg, user taps, button clicks, and mouse movements). Interaction trace log data generated by adolescent use of a DHBCI enables the collection of fine-grained information on user engagement. These data can be used to augment self-reported data, illuminating the processes and mechanisms of engagement with DHBCIs, as well as to drive AI models to flexibly support increased adolescent engagement in real time.

In looking at the future of DHBCIs, this paper presents AI-driven Mechanisms for Ethical Enhancement of Engagement (AIM-EEE), a framework for the design and implementation of DHBCIs that harnesses the potential of process-level data from adolescent interactions with technology and the analytical power of AI. AIM-EEE characterizes how AI techniques can be used to dynamically tailor DHBCIs to individual adolescents, deeply inform the design of DHBCIs, and provide analytics to care providers to further support and enhance care.

The AIM-EEE framework outlines 4 key roles for AI in DHBCIs for adolescent engagement, using measurement, modeling, optimization, and generation. The framework is situated in a call for increased attention to crucial ethical concerns that arise when using AI technologies, especially those related to privacy, algorithmic fairness, transparency, and accountability. We illustrate the framework using the example of INSPIRE [16], a narrative-centered behavior change environment designed to reduce adolescent alcohol use, to envision how AI can be leveraged to enhance adolescent engagement with DHBCIs and promote adolescent preventive health across domains of behavioral health. We conclude by arguing for more transdisciplinary research on the design and implementation of adolescent-specific DHBCIs that leverage the emerging capabilities of AI to support adolescent engagement and achieve the goal of improving adolescent health and well-being.

## Engagement in the Context of DHBCIs

Engagement is multidimensional, with cognitive, behavioral, affective, and social components [38,39]. Behavioral frameworks aiming to illuminate and measure multidimensional engagement with DHBCIs, such as Cole-Lewis and colleagues’ delineation of engagement with the intervention itself (“Little e”) and engagement in skills and behaviors the intervention aims to promote (“Big E”), provide a useful foundation [29-32]. However, such frameworks are not developmental, as they do not account for many rapidly evolving adolescent contextual and user attributes, which may not be fully captured in traditional self-report or behavioral measures [40-42].

In contrast to a one-size-fits-all approach to designing DHBCIs, AI-driven adaptivity aligns with the need for flexibility in accounting for adolescent engagement processes during a fluid phase of development. There is a natural alignment between the requirements of an adolescent-specific framework for engagement and the affordances of AI-driven technologies [19]. Specifically, AI technologies support personalization by applying machine learning techniques to measure user states and attributes [43,44], model patterns of user engagement [45], tailor responses to users’ needs and preferences [46,47], and generate entirely new content for interventions [48].

Several frameworks allude to the utility of AI and machine learning as logical next steps for measuring and analyzing in vivo engagement in DHBCIs, but they stop short of exploring how AI will serve in these roles and do not elucidate upon the issues raised by the introduction of AI technologies. To this end, computational frameworks have provided an initial roadmap for using AI to measure and support adolescent engagement with technology. For example, the AAA (advanced, analytic, and automated) approach is an AI-based framework for measuring engagement in educational technology [49]. This framework, which is grounded in theories of human cognition and affect, centers upon the use of machine learning to devise computational models for automatically inferring learners’ engagement states based on software log data, learner physiological data, and aspects of the environmental context. Although the AAA framework provides a clear account of how AI can be used to measure engagement, it does not address related tasks such as predicting learners’ future engagement, tailoring responses to enhance engagement, or generating newly engaging content, which are amenable to current and emerging AI technologies. Moreover, research on AI-based approaches for measuring and supporting user engagement in a health care context is limited. Rowe and Lester [33] identified promising AI technologies to support personalized preventive adolescent health care, including intelligent learning environments, interactive narrative generation, user modeling, and adaptive coaching. However, they do not address how foundational AI techniques and health care stakeholders fit together into a holistic picture to enhance understanding and support adolescent engagement with DHBCIs.

Taken together, these behavioral and computational engagement frameworks suggest that the vast magnitude and range of information afforded by process data (ie, interaction trace log data), combined with AI’s capacity to analyze and model such data, can contribute to a more nuanced and specific understanding of multidimensional adolescent engagement with DHBCIs and ultimately point to ways to enhance intervention effectiveness. Although applications of AI and machine learning can illuminate and enhance engagement in DHBCIs for adolescents, the lack of a comprehensive model means that the “how” remains unclear. The AIM-EEE framework synthesizes and fills gaps in previous work by providing a map of current (and prospective) roles and users of AI technologies in DHBCIs, as well as major flows of interaction between them, to support adolescent engagement in health behavior change.

## INSPIRE Digital Health Behavior Change Intervention

**Figure 1:**
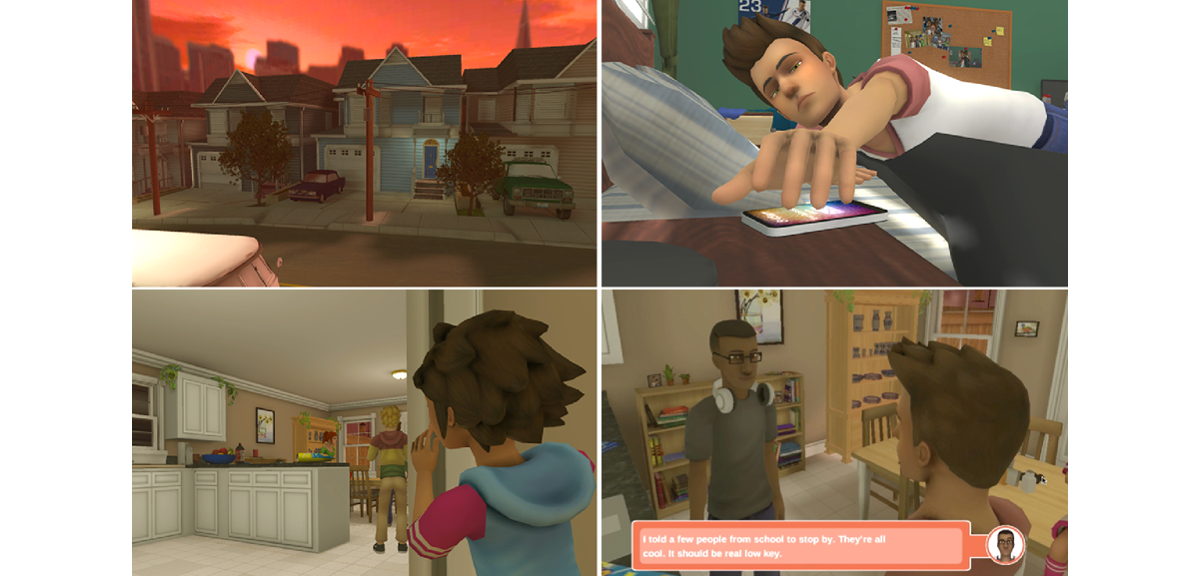
INSPIRE narrative-centered health behavior change environment.

Before we describe the AIM-EEE framework, we describe INSPIRE [16], a narrative-centered behavior change environment that is currently under development through a collaboration between the University of California, San Francisco, and North Carolina State University. INSPIRE will serve as an illustrative case to ground our discussion of AI-driven DHBCIs. INSPIRE is a narrative-centered health behavior change environment designed to help adolescents develop strategies for managing challenging situations involving alcohol use [16]. Adolescents are typically referred to the game through their primary care provider. When they begin the game, players take on the role of an adolescent protagonist, Max, reliving the events of a social gathering involving alcohol that goes awry (Figure 1). Over the course of multiple interactive narrative episodes, players encounter scenarios involving peer pressure, social norms, and alcohol-related consequences, enabling them to practice and build self-efficacy in handling risky health behaviors. As the episodes progress, the complexity of decisions and consequences surrounding alcohol use intensifies, further engaging and challenging players.

In a typical interaction with INSPIRE, the player starts by selecting goals for the evening for the character Max. The player’s choices, which may or may not align with their goals, influence the narrative as it dynamically unfolds. In each episode, players interact with a rich cast of characters, and the storyline takes place within a realistic 3D digital environment designed to feel authentic to adolescents. For example, in the first episode, the player is faced with decisions about inviting other teenagers, whom Max does not know, to come over to his house. The player must also respond to offers of alcohol from peers and make in-the-moment decisions about friends engaging in risky behavior involving alcohol use. In the second episode, the player makes decisions about navigating the social gathering after it has spiraled out of Max’s control, including situations that involve property damage, binge drinking, and a discussion about driving under the influence of alcohol.

INSPIRE is based on social-cognitive theory for behavior change, with a key focus on increasing self-efficacy [16,50,51]. It is hypothesized that adolescent users’ perceived self-efficacy can be enhanced by vicariously experiencing and practicing handling challenging scenarios relating to alcohol use through relatable avatars. Importantly, INSPIRE is designed to serve as a clinician-extender, facilitating behavior change outside of the clinic, as well as a rich source of cross-domain information on adolescent engagement. See prior work for an in-depth description of INSPIRE [16].

In addition to being a DHBCI for adolescent preventive health, INSPIRE serves as a research test bed for investigating novel AI technologies that promise to enhance understanding of adolescent engagement and support adolescent health behavior change, including through the collection of interaction trace log data. In INSPIRE, the time-stamped logs show how long users spend at decision-points, converse with digital characters, interact with in-game objects, and select and enact goal-oriented behavior, as well as which decisions they make. In addition to enriching insights gleaned from users’ self-reported information, these data can also be used to drive the training, testing, and operation of AI models designed to enhance adolescent engagement in the DHBCI.

## AIM-EEE Framework

### Overview

**Figure 2:**
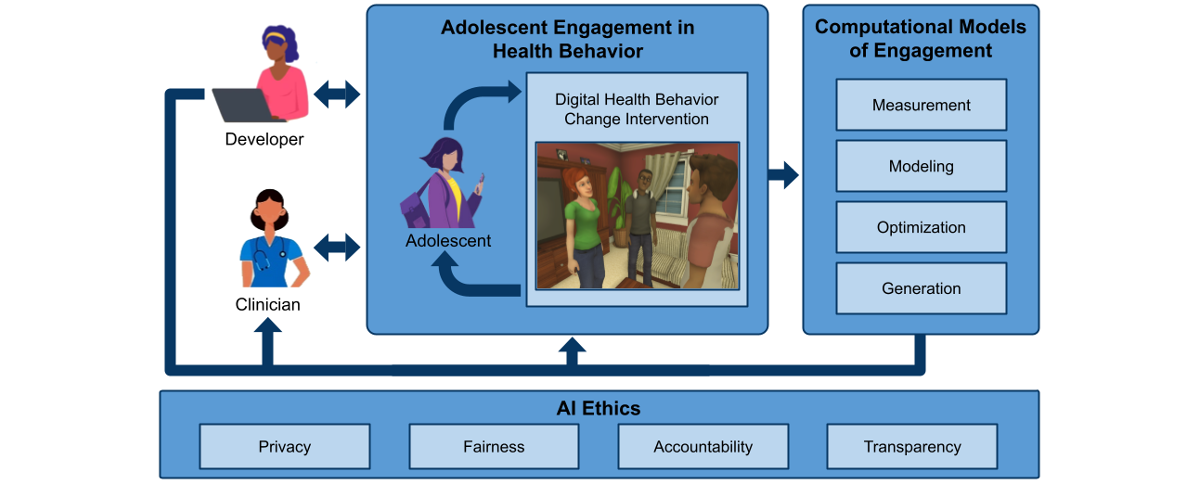
The AI-Driven Mechanisms for Ethical Enhancement of Engagement (AIM-EEE) framework for the design, development, and implementation of digital health behavior change interventions. AI: artificial intelligence.

In this section, we introduce the AIM-EEE framework for the design and implementation of AI-based DHBCIs to support adolescent engagement in health behavior change (Figure 2). We first describe the four key roles of AI technologies in adolescent engagement with DHBCIs: (1) measuring adolescent engagement, (2) modeling adolescent engagement, (3) optimizing adolescent engagement, and (4) generating engaging content. These roles are identified by drawing upon the empirical literature on AI-based technologies in other domains, including education [52,53], military training [54], and entertainment [36]. We describe ways that adolescents, clinicians, and system designers can use AI technologies to support adolescent engagement in DHBCIs and health behavior change more broadly. We then discuss the important challenges and issues raised by AI-driven DHBCIs, which point toward the need for establishing standards and best practices for ethical AI approaches that are infused throughout this type of work. Finally, we raise questions regarding how these findings are likely to translate to the adolescent preventive health care domain and discuss key factors that may support, or potentially limit, their generalizability.

### Measurement

Machine learning enables automated measurement of adolescent engagement during user interactions with DHBCIs. Interaction trace log data can be analyzed using machine learning techniques to detect different facets of user engagement, including cognitive [44,55-57], affective [49,58,59], behavioral [60-64], and social [65-67] components. Machine learning techniques have been applied to measure a broad range of engagement phenomena, including goal setting [68], learning [44], problem-solving [69], affect [49], and reflection [65] among others. In addition, there has been significant attention toward using machine learning to measure disengagement with technology [63].

An important requirement of many machine learning techniques is the availability of “labeled” data, or in this case, data that has been annotated with example measurements of adolescent engagement to help train the AI system. *Supervised learning* is a family of machine learning techniques that uses labeled data to train models for predicting the output associated with each input data point [70]. When developing a computational model to measure user engagement with supervised learning, the training data is annotated with engagement labels, which are typically obtained using self-report or observational methods. By automating the process of assigning labels to new, unlabeled data, supervised learning creates opportunities for automating the measurement of user engagement, identifying the signatures of cognitive, affective, behavioral, or social engagement that can be automatically captured and recognized in interaction trace log data. In comparison to traditional measures, machine learning approaches offer distinctive affordances in terms of scalability and minimal obtrusiveness, which are attractive for studying and fostering adolescent engagement with DHBCIs.

To develop a machine learning model for measuring adolescent engagement with a DHBCI such as INSPIRE, variables are extracted from adolescents’ raw trace log data to serve as indicators of adolescent engagement with INSPIRE (Little E Engagement). These variables, also known as *features*, serve as input to machine learning algorithms. In INSPIRE, features may include the choices adolescents make while conversing with digital characters, the types of goals that an adolescent sets for their avatar in the digital environment, or the frequency and duration of adolescent interactions with in-game objects, among others. Machine learning models can be trained to analyze these features and predict different components of engagement (eg, interest and affect), initially measured using external instruments (eg, questionnaires and field observations) that generalize new users and situations. Machine learning models can be devised that provide coarse-grained measurements of engagement, such as a single measurement per adolescent who has interacted with the system, or more fine-grained measurements that track adolescents’ moment-to-moment engagement levels with the technology. These methods have been widely used to measure adolescent knowledge [55], affect [58], and self-regulatory processes [71] in narrative-centered learning environments for K-12 education, and their generalizability to DHBCIs such as INSPIRE is a key open question for the field. Notably, in adolescent health care, there are many opportunities for introducing both self-report measurements (eg, responses to questions about quantity and frequency of past 30-day alcohol use) and objective measurements (eg, levels of ethyl glucuronide, a byproduct of alcohol, which can remain in the urine for up to 5 days after alcohol consumption) to train AI-based models for assessing adolescent engagement in real-world behavior change (Big E engagement). These opportunities are also not limited to adolescent health behavior change related to alcohol. For example, for a behavioral intervention targeting weight loss, wearable devices measuring activity, sleep, and other indicators of behavior change, as well as biometric indicators of obesity-related health markers, could be used to enhance DHBCI diagnostics and insights.

### Modeling

A key function of machine learning is to create computational models that capture patterns in large, complex data sets. Computational models can serve several purposes, such as making future predictions about adolescent engagement as well as examining engagement-related phenomena in greater detail. Machine learning models of adolescent engagement can take several different forms. Predictive models of adolescent engagement can be trained to forecast both short-term and long-term engagement behaviors in the future. For example, in education, machine learning has been used to make early predictions about how long a visitor will engage with a game-based museum exhibit [72]. Machine learning has also been used to predict students’ retrospective self-reports of interest in a narrative-centered learning environment collected shortly after interacting with the system [73]. As far as long-term prediction, machine learning has been used to create predictive models that analyze middle school students’ interactions with an intelligent tutoring system to predict college enrollment decisions and Science, Technology, Engineering, and Mathematics career interests many years in the future [74-76]. Similarly, machine learning has been used to predict student dropouts from school [61,77,78]. These models may require collecting longitudinal data on adolescent behavior to obtain labels for training engagement models using supervised learning techniques. Many of these predictive models focus on engagement constructs analogous to Cole-Lewis and colleagues’ “Big E” engagement [30]. In other words, the predictive models are not only measuring engagement with the intervention (eg, INSPIRE) but also engagement with the health behavior itself (eg, reduction in alcohol use). Like the work on AI-based measurement of engagement, the generalizability of AI-based predictive modeling from education to adolescent health behavior change is a key research question.

Machine learning techniques can also be used to uncover complex patterns in trace log data, discovering new forms of engagement behavior that are only apparent through data-driven analysis of adolescent interactions with the technology. An important family of machine learning techniques for discovering patterns in interaction trace log data is *unsupervised learning.* Unsupervised learning techniques, such as cluster analysis, do not require labeled data and can reveal unexpected patterns in user behavior [79]. For example, unsupervised learning techniques have been used to uncover different strategies that youths engage in when interacting with digital technologies, including strategies that may not have been predicted from the literature [80]. Another technique that recognizes sequential patterns of unfolding behavior is called differential sequence mining, which can be used to distinguish different groups of users, such as high- and low-engagement users [81].

In the example of INSPIRE, investigating the creation of predictive models of adolescent engagement in health behavior (ie, Big E engagement and reduced alcohol use) with machine learning techniques is a promising future direction. Similarly, applying cluster analysis to adolescent interactions with INSPIRE could reveal different strategies in how teens engage with digital characters, interact with digital objects, set goals, and self-regulate while using DHBCIs.

### Optimization

A third use case for AI is incorporating dynamic personalization functionalities into a DHBCI to improve the overall effectiveness and enhance adolescent engagement. Specifically, adolescent interactions with DHBCIs can be algorithmically optimized by adapting how, when, and what type of interventions are delivered. A family of machine learning algorithms that has received considerable attention for this type of task is *reinforcement learning*. Reinforcement learning refers to a broad range of techniques for training a software agent to make sequential decisions in an uncertain environment to optimize (potentially delayed) reward [82]. This family of algorithms has been used in domains such as automated game-playing [83], robotic control [84], and autonomous vehicles [85]. It has also been applied toward more human-centered tasks, such as intelligent tutoring systems [86], player-adaptive games [87], and increasingly, health interventions [88]. In this latter category of tasks, reinforcement learning has been used to drive pedagogical decisions about how to support student problem-solving in adaptive learning environments [47,86], guide the behavior of nonplayer agents in computer games [89], and optimize coaching strategies and behavior change interventions for obesity [88]. Reinforcement learning techniques have also been used to optimize personalized coaching interventions for health behavior change in domains such as weight loss and physical activity [88]. For example, Forman et al [88] investigated a reinforcement learning–based weight loss intervention that involved twice-weekly remote interventions consisting of phone calls, text exchanges, and automated messages. Results indicated that the reinforcement learning–optimized intervention was feasible to deploy, acceptable to participants and coaches, and achieved equivalent weight losses as alternative interventions at a significantly reduced cost. The findings highlight the promise of using reinforcement learning and AI techniques more broadly for the optimization of DHBCIs for obesity and other health behavior change domains.

A range of factors can inform AI-driven decisions about how to optimize components of a DHBCI at runtime, including adolescents’ traits and interactions with the technology-based intervention. Similarly, many different variables can be used to serve as the optimization criteria for runtime adaptation. In INSPIRE, reinforcement learning techniques may seek to tailor the intervention to optimize for reducing self-reported alcohol use, improving self-efficacy to avoid risky behaviors, or even increasing knowledge about alcohol and its effects. Reinforcement learning can also be used to develop policies for optimizing a range of different components of a DHBCI. In an intervention such as INSPIRE, reinforcement learning can be used to guide the behaviors of a positive role model character in the interactive narrative. Alternatively, reinforcement learning can be used to devise models for selecting and tailoring scaffolds that are delivered at runtime to enhance the intervention’s effectiveness. Decisions about introducing, adapting, or removing narrative vignettes, which represent practice opportunities for navigating challenging situations about risky behavior and alcohol use in INSPIRE, can be dynamically sequenced and tailored using reinforcement learning–based models.

### Generation

Recent years have seen dramatic advances in generative AI based upon deep learning techniques to produce synthetic images, videos, and text that can be used for a range of functions and applications with relevance to the design and development of DHBCIs. For example, generative adversarial networks have proven to be a powerful algorithmic approach for creating synthetic images by training on an existing corpus of image data in order to produce new images that are difficult to distinguish from actual images [90]. Similarly, striking advances in large language modeling techniques such as transformers [91] and ChatGPT [92] have yielded powerful capabilities for producing text that resembles human-authored material, both in natural language and other forms. Research on narrative generation has investigated methods for automatically creating the plot structures and cinematic techniques involved in producing compelling narrative media [93,94]. Research on procedural content generation has focused on using a range of algorithmic techniques to produce digital environments that users can explore and engage with within the context of narrative and nonnarrative games [95].

These methods can be leveraged to automatically generate the individual components that compose narrative-centered behavior change environments such as INSPIRE. For example, natural language generation techniques can be used to automatically synthesize the dialogue for digital characters in an environment like INSPIRE so that it is contextually appropriate. Procedural content generation techniques can be used to automatically construct the digital environments in which the interactive narrative events that adolescents encounter in an environment like INSPIRE to ensure that they are novel and relevant. The progression of story events can be based upon dynamically synthesized story lines that have been generated by a model trained from corpora of previous stories. Although still in their nascent stages, these methods show significant promise in terms of their capacity to promote novel story-based learning experiences in narrative-centered behavior change interventions, as well as tailor the design of the narratives to enhance relatability to developmentally and demographically diverse teens, which are both important factors in fostering engagement and supporting health behavior change in adolescents.

## Special Considerations for AI-Driven DHBCIs

### Who Stands to Benefit?

It is essential to consider the perspectives of different health care stakeholders in the design and implementation of AI-driven DHBCIs. Adolescent interactions with DHBCIs are central to the framework. The resulting data can be used to drive AI-based models of adolescent engagement. From an adolescent’s perspective, AI models enable personalized health behavior change interventions that are dynamically tailored to the adolescent at runtime. Indeed, adolescents stand to be the primary beneficiaries of AI technologies in DHBCIs.

DHBCI developers will also benefit from AI technology developments in several important ways. The data-driven insights yielded by machine learning techniques can often be translated into design implications to inform the creation and refinement of DHBCIs [56,71,94]. Generative AI tools also show promise for supporting developers in the creation of AI-driven DHBCIs, creating new possibilities for how developers actually create DHBCIs through AI-augmented developer tools [96] and simulated user functionalities [87].

For clinicians, the prospective role of AI in the automated measurement and modeling of adolescent engagement creates opportunities for generating analytics on how adolescents engage with DHBCIs. Clinicians can use analytics to inform the care they provide and monitor how patients engage with an intervention. An important consideration is how analytics fit into clinical workflows to best meet the needs of care providers and their patients. For example, a care provider who examines engagement analytics from an adolescent patient before their annual checkup might use this information to inform a discussion they have with the patient during the visit about different types of health behavior, potential forms of risk, and preventive strategies that promote adolescent health. These types of analytics also promise to enrich what care providers might know about their adolescent patients’ health behaviors, which is necessarily limited by the relatively brief duration of visits with patients. In effect, AI technologies provide an opportunity for extending the reach of clinicians, enriching their capacity to connect with and assess their patients, and expanding the types of behavior change interventions possible through the combination of technology and care providers.

### Ethical AI in DHBCIs

While the enhancement of DHBCIs with AI and machine learning holds promise, it is also crucial to recognize new and rapidly evolving ethical quandaries. Ethical use of AI and machine learning becomes even more vital as we turn our attention to enhancing engagement in DHBCIs [97]. In light of these quandaries, the AIM-EEE framework is situated within the foundational ethical principles of privacy, algorithmic fairness, transparency, and accountability.

As AI algorithms are strengthened by an increasing volume and variety of data, researchers are incentivized to use inconspicuous methods of collection that maximize both. The collection of data in a “stealth” manner, outside of the user’s awareness after initial consent or assent, in adolescents, is made even more complex by the requirement for the dual education of both minor study users and their guardians, especially in web- or app-based research where a parental consent checkbox may be one that the adolescent themselves can click. Therefore, it is especially important to ensure that the research community is thoughtful about informed consent and user privacy standards while gathering data on this population [33]. Existing evidence suggests that both adolescents and adult users often agree to stealth data collection without a full understanding of privacy implications [98]. Moreover, adolescents tend to report more concern for and confidence in regulating their social privacy than institutional privacy (eg, use of their data by a government, corporation, or research entity) [99,100], rendering them particularly vulnerable to exploitation in this context [19,101].

There is also growing recognition of the importance of fairness and encoded bias in AI systems. Special attention must be paid to the characteristics of the developers, the data on which it was built, and how fairly AI algorithms perform. It is well established that biased data inputs will produce biased models, so a nuanced understanding of how to measure and mitigate encoded bias will be vital to ethical AI [102]. This will involve including diverse populations in DHBCI and AI model development, validation, and data collection not only to decrease the risk of bias but also to broaden our understanding of how engagement with DHBCIs varies across demographically and developmentally varied populations. This is particularly important for adolescents from groups historically underrepresented in or exploited by health research [103-105]. Researchers risk underrepresenting large swaths of adolescents and thus creating biased AI models if they do not first proactively solicit and act on these communities’ concerns about stealth data privacy, especially as more research moves digitally. For stealth data collection with adolescents to be truly inclusive, useful, and ethical, the research community must establish protocols for privacy and informed consent with stealth data, along with an accountability infrastructure to undergird it.

Finally, transparency is critical at all stages of the AI-driven DHBCI life cycle, including the design of AI algorithms, the selection of tasks that AI systems are directed to solve, the training and testing of AI models, and the practical use of AI systems in real-world health care settings. Devising ethical standards for reporting highly consequential variables or the roles of distinct algorithmic processes with the intention of providing a broader and deeper understanding of how AI systems operate will be critical [106]. Researchers and research users must jointly create systems of accountability that set standards for privacy, fairness, and transparency. Without accountability, these ethical ideals may remain just that.

## Limitations and Future Directions

Many of the roles for AI that are outlined in the AIM-EEE framework are based on the extant literature on AI applications in related domains. There is compelling evidence suggesting that AI systems can operate effectively across a range of different roles for supporting adolescent learning and engagement. Furthermore, transferring AI techniques from one domain to another, for example, from education to adolescent health behavior change, is central to the enterprise of AI research. Yet, human behavior related to health presents its own distinct sources of complexity and uniqueness. In turn, the aim of purposing AI techniques toward supporting adolescent engagement in health behavior change introduces myriad open questions and challenges that merit investigation by the broader research community. This assumption that AI techniques for modeling adolescent engagement will successfully generalize health behavior change is an important limitation of this work. There is a significant need for greater research by transdisciplinary teams of computer scientists, health care researchers, behavior change experts, intervention designers, and health care practitioners to demonstrate how to maximize this generalization to different areas of adolescent health behavior change. Developing increased research capacity and supporting new research programs to conduct this type of work is likely to become imperative for the field to ensure the responsible and effective use of AI technologies for supporting adolescent engagement in health care settings. Finally, there are many important ethical considerations to be mapped out and accounted for in the usage of AI systems intersecting with the collection and analysis of health data, particularly with adolescents.

## Conclusions

The AIM-EEE framework is positioned to support researchers, developers, and providers in understanding and leveraging adolescent engagement in DHBCIs. AIM-EEE is rooted in the automated, machine learning–based analysis of trace log data that is readily generated during adolescent interactions with DHBCIs. Log data serves to drive AI and machine learning techniques to train and validate models that recognize patterns of user behavior. These techniques enable the examination of associations between self-reported engagement and in-game behaviors, as well as a more holistic analysis of factors that may impact user experience and engagement with both the intervention itself and the health behaviors the intervention aims to promote on a range of time scales. This can be harnessed for personalization and dynamic tailoring of future user interactions, which is particularly crucial for the fluid development characteristic of adolescence, ultimately increasing the efficacy of interventions such as INSPIRE through enhanced engagement. Importantly, the use of these powerful computational tools and techniques with youths must be grounded in a vision of ethical AI, with attention to the myriad privacy and accountability pitfalls inherent to the use of AI at the forefront of innovation.

## Abbreviations

AAA: advanced, analytic, and automated
AI: artificial intelligence
AIM-EEE: AI-driven Mechanisms for Ethical Enhancement of Engagement
DHBCI: digital health behavior change intervention
